# Enhanced Bioavailability of Tadalafil after Intranasal Administration in Beagle Dogs

**DOI:** 10.3390/pharmaceutics10040187

**Published:** 2018-10-15

**Authors:** Jeong-Soo Kim, Min-Soo Kim, In-hwan Baek

**Affiliations:** 1 Dong-A ST Co. Ltd., Giheung-gu, Yongin, Gyeonggi 446-905, Korea; ttung2nd@gmail.com; 2 College of Pharmacy, Pusan National University, Busan 46241, Korea; 3 College of Pharmacy, Kyungsung University, 309 Suyeong-ro, Nam-gu, Busan 48434, Korea

**Keywords:** tadalafil, pharmacokinetics, intranasal, modeling, dog

## Abstract

Tadalafil is an oral selective phosphodiesterase type-5 inhibitor with demonstrated efficacy and safety that is used to treat erectile dysfunction. The purpose of this study is to compare the pharmacokinetic properties of tadalafil after conventional oral tablet administration and novel intranasal administration in beagle dogs. Fourteen 13-month-old male beagle dogs were randomly divided into two groups, and were given 5 mg tadalafil orally or intranasally in a parallel design. Blood samples were collected before and 0.5, 1, 1.5, 2, 4, 6, 8, 12, 24, and 36 h after administration. The plasma concentration of tadalafil was determined via liquid chromatography-tandem mass spectrometry (LC-MS/MS). The systemic exposure and absorption rate of tadalafil were significantly greater in the intranasal administration group than in the oral administration group. A one-compartment model with first-order absorption and elimination was sufficient to explain the pharmacokinetic characteristics observed after both oral and intranasal administration. This study indicates that the development of tadalafil nasal delivery systems is feasible and may lead to better results than the conventional oral route.

## 1. Introduction

Tadalafil is a selective inhibitor of phosphodiesterase type-5 (PDE5), an enzyme that inactivates cyclic guanosine monophosphate (cGMP), and has demonstrated efficacy and safety as an oral therapy for erectile dysfunction (ED) [1,2]. Furthermore, tadalafil has a greater selectivity for PDE5 than sildenafil, the first approved PDE5 inhibitor, and one of the most widely used PDE5 inhibitors worldwide [3]. Tadalafil also has a prolonged half-life, with a low volume of distribution, slow hepatic clearance, and approximately 80% bioavailability in human. The pharmacokinetic properties of tadalafil facilitate a prolonged duration of action through once-daily dosing, so that sexual spontaneity may be more easily restored [4].

The original formulation of tadalafil was released in 2003 as a film-coated tablet for oral administration [5]. However, this formulation has been inconvenient for patients, as it must be swallowed with water. More importantly, as ED is frequently associated with depression, increased anxiety, poor self-esteem, and compromised interpersonal relationships [6], ED patients require a treatment that has a rapid onset and a long half-life, allowing for easy administration.

In order to meet patients’ needs, various formulations have been investigated, including orodispersible formulations, orally disintegrating film formulations, and transdermal patches [5,7,8,9,10,11]. In addition, the pharmacokinetics of sildenafil and udenafil have been investigated, following intranasal administration in animals [12,13]. Relative to oral administration, intranasal administration has several advantages, including a fast onset of effectiveness due to rapid absorption, avoidance of intestinal and hepatic first-pass effects, greater bioavailability allowing for lower doses, a reduction in gastrointestinal disturbances, a reduced risk of overdose, non-invasive administration, ease of convenience and self-medication, and improved patient compliance [14,15,16]. However, pharmacokinetic and formulation studies of tadalafil following intranasal administration have not yet been performed.

Thus, the purpose of this study was to compare the pharmacokinetic properties of tadalafil after conventional oral tablet administration and novel intranasal administration in beagle dogs. The pharmacokinetic parameters of tadalafil were obtained via noncompartmental analysis and modeling. This study furthers the possibility of intranasal tadalafil administration as a novel drug delivery system.

## 2. Materials and Methods 

### 2.1. Chemicals and Reagents

Tadalafil and sildenafil citrate (internal standard—IS) were purchased from Sigma Chemical Co. (St. Louis, MO, USA) for use in liquid chromatography-tandem mass spectrometry (LC-MS/MS). High-performance (HP)LC-grade acetonitrile and methanol were purchased from Burdick and Jackson (Muskegon, MI, USA). All of the other chemicals and solvents were of the highest analytical grade available. Cialis^®^ tablets containing 5-mg tadalafil were purchased from Lilly Korea Co., Ltd. (Seoul, Korea).

### 2.2. Animal Study

Fourteen 13-month-old male beagle dogs weighing 9.19–12.27 kg were provided by Woojungbio., Co., Ltd. (Suwon, Korea) and were kept individually in controlled environments at a temperature of 23 ± 2 °C and a 12/12 h light/dark cycle for a two-week acclimatization period. A quantitative pellet diet was given at a fixed time each day, and water was offered ad libitum. All of the physical examinations and blood tests were acceptable for use in the experiments.

The animal experiments were conducted in accordance with the Guidelines for the Care and Use of Laboratory Animals, and were approved by the Institutional Review Board of the nonclinical contract research organization, KPC laboratory (approval date: 29 September 2017). The fourteen dogs were randomly divided into two groups. The dogs in group A (*n* = 7) were administered tadalafil 5 mg (Cialis^®^ 5 mg) orally in the morning after an overnight fast. The dogs in group B (*n* = 7) were administered tadalafil 5 mg intranasally using a manual pump spray unit that delivered 120 μL of the formulation per spray. The ingredients of the nasal spray formulation were as follows: 30% polyethylene glycol (PEG) 400, 50% transcutol HP, and 5% Tween 80 in normal saline. A total volume of 240 μL formulation was sprayed into both of the dogs’ nostrils at a dose of 5 mg for each dog. The dogs’ heads were elevated for approximately 30 s during the administration, and for approximately 30 s after the administration. No food or water was allowed until 4 h and 2 h after administration, respectively. The blood samples (approximately 1.5 mL) were deposited into heparinized tubes before (0 h) and 0.5, 1, 1.5, 2, 4, 6, 8, 12, 24, and 36 h following the drug administration. All of the blood samples were centrifuged for 2 min at 10,000 rpm (13,416 × *g*), and the plasma was stored at −70 °C until the HPLC-MS/MS analysis.

### 2.3. Determination of Tadalafil Concentration in Plasma Using LC-MS/MS

The plasma concentrations of tadalafil were quantified via LC-MS/MS using an Agilent 1260 series (Agilent Technologies, Santa Clara, CA, USA) and API 2000 MS/MS system (Applied Biosystems, Foster City, CA, USA), equipped with an electrospray ionization interface to generate positive ions [M − H]^+^. An HPLC chromatographic separation was performed on a Zorbax SB C_18_ column (50 × 4.6 mm, 5 μm) with Phenomenex SecurityGuard Cartridges (C_18_, 4.0 × 3.0 mm, Macclesfield Cheshire, U.K.). The mobile phase composition was a mixture of acetonitrile—10 mM ammonium formate buffer (70:30, *v*/*v*, pH 3.0 with formic acid) at a flow rate of 300 μL/min. The column and autosampler were set at 30 and 10 °C, respectively. The analytes were detected using the multiple-reaction-monitoring (MRM) mode at transitions of *m*/*z* 390.1→268.2 for tadalafil and *m*/*z* 475.3→100.0 for the IS. The collision energy, and declustering and collision exit potentials were set to 17, 76, and 12 V for tadalafil, and 31, 91, and 10 V for the IS. The ion spray voltage and entrance potential were set to 5500 and 10 V, respectively. The dwell time was 150 ms for the analytes. The data were processed using the Analyst 1.4.1 software. 

The tadalafil and the IS were extracted from the plasma matrix via protein precipitation. In an Eppendorf tube^®^, 600 μL of acetonitrile containing the IS (500 ng/mL) was added to a 100 μL-plasma sample. After vortex mixing and centrifugation at 12,000 rpm for 5 min, an aliquot of supernatant (300 μL) was transferred to an autosampler vial, and 5 μL of the sample was injected into the LC-MS/MS system. The method validation was carried out according to the United States Food and Drug Administration Bioanalytical Method Validation Guidance [17], and the linearity for tadalafil was achieved between 1–1000 ng/mL. The intra- and inter-day precisions (*n* = 5) of the assay were 4.2–11.8%, and the intra- and inter-day accuracies (*n* = 5) were 89.7–110.2%. The short-term (room temperature for 6 h), post-extraction (4 °C for 24 h), freeze–thaw (−70 °C after three cycles), and long-term stabilities (−70 °C for 1 month) were adequate. The pharmacokinetic samples were conducted using the same procedure.

### 2.4. Noncompartmental Pharmacokinetic Analysis

The noncompartmental pharmacokinetic analyses were evaluated using WinNonlin Standard Edition software, version 5.2 (Pharsight Corp., Mountain View, CA, USA) [18]. The area under the plasma concentration versus time curve from 0 to 36 h (*AUC*_36h_) was assessed using the linear trapezoidal methodology, and was extrapolated to infinity (*AUC_inf_*). The maximum plasma concentration (*C_max_*) and time to reach *C_max_* (*T_max_*) for tadalafil were directly obtained from the individual observed data. The terminal phase elimination rate (*λ_z_*) was estimated using a log-linear regression of the observed plasma concentration point in the terminal phase, and the elimination half-life (*t*_1/2_) was calculated as 0.693/*λ*_z_. The apparent total clearance (*Cl_t_/F*) and volume of distribution (*V_z_/F*) were calculated using the formulas *dose/AUC_inf_* and *dose/(K_el_*·*AUC_inf_*), respectively.

### 2.5. Pharmacokinetic Modeling Analysis

A one-compartment pharmacokinetic model with first-order absorption and elimination rate constants was applied to describe the pharmacokinetic profiles of tadalafil after oral and intranasal administration in dogs. Two differential equations were consisted to explain the changes in the amount of tadalafil between the depot and central compartments after oral and intranasal administration, namely:

*dA*(1)/*dt* = −*K_a_* × *A*(1), (1)

*dA*(2)/*dt* = *K_a_* × *A*(1) − *K_el_* × *A*(2), (2)

where *A*(1) and *A*(2) indicate the amounts of tadalafil in the depot and central compartments, respectively; *K_a_* is the first-order absorption rate constant for tadalafil from the depot to the central compartment; and *K_el_* is the first-order elimination rate constant for tadalafil. The differential equations were fitted to the dataset using the maximum likelihood expectation maximization (MLEM) algorithm in ADAPT 5 (Biomedical Simulation Resource, Los Angeles, CA, USA) [19]. The data below the quantification limit (BQL; <1 ng/mL) were excluded from the analysis.

The plasma concentrations of tadalafil (*C_p_*) after oral and intranasal administration were calculated using the following equation:

*C_p_* = *X*(2)/(*V_c_*/*F*), (3)

where *V_c_/F* is the volume of distribution in the central compartment. The equations were applied to the data using ADAPT 5, under the assumption that the standard deviation of the measurement error was a linear function of the measured quantity (Var[*ε*_i_(*t*_i_)] = (*σ*_0_ + *σ*_I_*C*(*t*_i_))^2^). The model evolution was conducted using the goodness of fit, Akaike’s information criterion (AIC), Schwartz’s Bayesian information criterion (SC), the sum of squares of the residuals, visual examination of the distribution of residuals, log-likelihoods, coefficients of variation of parameter estimates, and parameter correlation matrices [20].

### 2.6. Statistical Analysis

The data are represented as the mean ± standard deviation. The differences in the noncompartmental model pharmacokinetic parameters of tadalafil between the oral and intranasal administrations were tested using the student *t*-test. The statistical significance was assigned to *p*-values less than 0.05. All of the statistical analyses were conducted using SPSS software (version 20.0; SPSS, Chicago, IL, USA).

## 3. Results

### 3.1. Noncompartmental Pharmacokinetic Analysis

**Figure 1:**
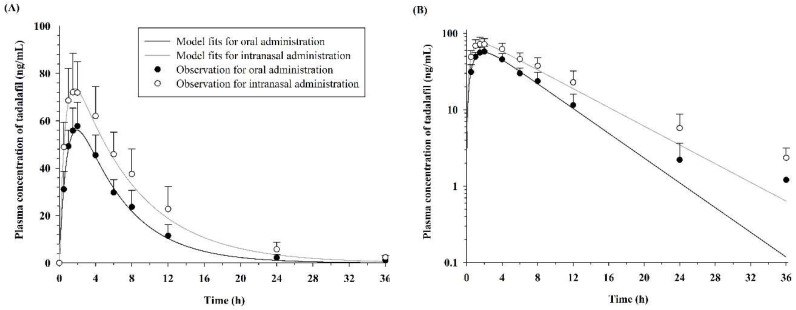
Mean plasma concentration–time curves (**A**) and the corresponding graph converted to a semi-log scale (**B**) for the oral and intranasal administration of 5 mg tadalafil in dogs. Each point represents the mean ± standard deviation (SD). Solid lines indicate the final model fits.

The plasma concentration versus time profiles of tadalafil after the oral and intranasal administration of a 5 mg dose are shown in Figure 1, and the corresponding noncompartmental pharmacokinetic parameters are listed in Table 1. Following the oral administration, the maximum concentration of tadalafil was 59.49 ± 9.22 ng/mL at 1.71 ± 0.39 h, and the *AUC*_36*h*_ and *AUC_inf_* were 472.66 ± 102.70 ng·h/mL and 479.33 ± 102.88 ng·h/mL, respectively. Following intranasal administration, the maximum concentration of tadalafil was 76.45 ± 12.07 ng/mL at 1.50 ± 0.41 h, and the *AUC*_36*h*_ and *AUC_inf_* were 771.10 ± 216.72 ng·h/mL and 790.23 ± 224.91 ng·h/mL, respectively. Compared with the oral administration group, the intranasal administration group exhibited significantly greater values for *C_max_*(1.29-fold, *p* < 0.05), *AUC*_36*h*_ (1.63-fold, *p* < 0.01), and *AUC_inf_* (1.65-fold, *p* < 0.01). However, there was no significant difference in the *T_max_* values between the oral and intranasal administrations.

### 3.2. Pharmacokinetic Model Analysis

**Figure 2:**
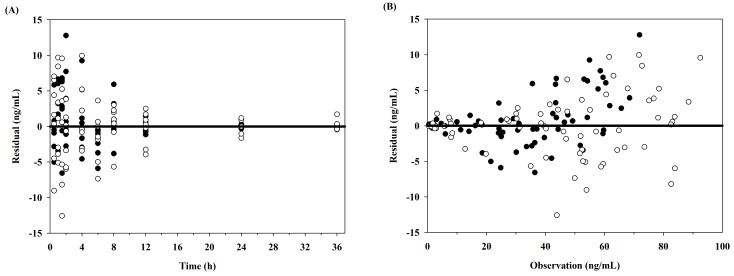
Diagnostic plots obtained from the final pharmacokinetic model of tadalafil after oral (closed circles) and intranasal administration (open circles) in dogs. (**A**) Residuals versus time after dose; (**B**) residuals versus observed concentrations.

A one-compartment pharmacokinetic model with first-order absorption and elimination rate constants successfully described the pharmacokinetic properties after oral and intranasal administration of 5 mg tadalafil to dogs. In Figure 1, the solid lines and circles show the one-compartment pharmacokinetic model fits and observations, respectively, of the average plasma concentration–time profiles for tadalafil. The diagnostic plots of the residuals versus time and residuals versus observation data are shown in Figure 2. In addition, the final estimates for the model parameters are listed in Table 2.

Among the model parameters, the value of *K_a_* for the intranasal administration of tadalafil was significantly greater than that for the oral administration (*p* < 0.001). Moreover, the value for *V_c_/F* for the intranasal administration of tadalafil was significantly lower than that for the oral administration (*p* < 0.05). However, there was no significant difference in the values of *K_el_* after oral and intranasal administration.

## 4. Discussion

In this study, the pharmacokinetic properties of 5 mg oral and intranasal tadalafil were compared after administration in dogs. The pharmacokinetic parameters of tadalafil were obtained via noncompartmental analysis and pharmacokinetic modeling analysis. The pharmacokinetic exposures following the intranasal administration of tadalafil were statistically greater than those following oral administration. In particular, the absorption rate was statistically faster and the bioavailability was statistically greater for the intranasal administration relative to the oral administration, based on the modeling analysis.

The pharmacokinetics of the PDE5 inhibitors using various administration routes have previously been assessed in animals and humans [5,7,8,9,10,12,13,21]. Particularly, the intranasal delivery of PDE5 inhibitors has been highlighted because of its advantages [22]. Indeed, the pharmacokinetics of sildenafil and udenafil have been examined in previous studies. Until now, however, there have been not yet been any studies of tadalafil in animals or humans. 

Previous studies of sildenafil and udenafil showed shorter *T_max_* values and a higher exposure for the intranasal versus oral administration [12,13,21]. Similarly, in this study, the pharmacokinetic tadalafil exposure after intranasal administration was significant higher than after oral administration. However, *T_max_* did not differ significantly between intranasal and oral administration. This may be due to the fact that the *T_max_* for tadalafil after oral administration was already short, so the faster absorption found in intranasal drug delivery might not affect *T_max_*.

**Figure 3:**
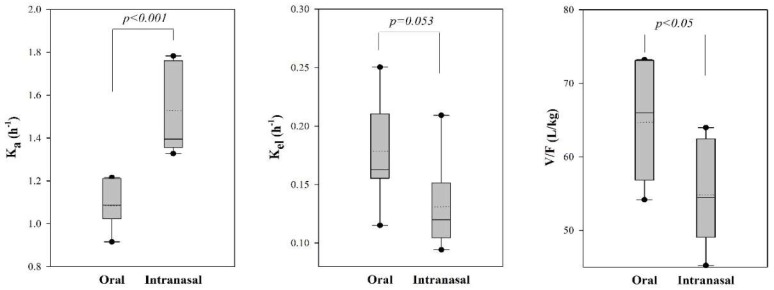
Box plot of model parameters for tadalafil after oral and intranasal administration in dogs (*n* = 7/group). The median is displayed as (−) and the mean as (···). Boxes are drawn from 25th to 75th percentiles, and whiskers extend from 5th to 95th percentiles. Circles lie outside the range of the 5th to 95th percentiles.

The results of the modeling approaches were consistent with a noncompartmental analysis (Figure 3). The absorption rate constant (*K_a_*) was greater following the intranasal administration versus oral administration in dogs. Further, the value of *V_c_/F* in the intranasal administered group was smaller than that for the oral administered group. The difference in *V_c_/F* might be caused by the increased bioavailability (*F*) in the intranasal group, and might not be affected on distribution. The elimination rate constant (*K_el_*) was smaller in the intranasal group, but this finding did not reach statistical significance (*p* = 0.053). These results suggest that the intranasal administration of tadalafil led to a faster and greater absorption than the oral administration.

A dog model is useful for the assessment of pharmacokinetic parameters for human scale dosing [23]; however, there are no previous studies that examine the pharmacokinetics of tadalafil in dogs, except for the pharmacology review of Cialis^®^ for U.S. FDA approval, and we determined to undertake the analysis. There are some differences in the pharmacokinetic parameters of tadalafil between dogs (*t*_1/2_ = 4.17 h; *F* = 10–18% for oral administration) and humans (*t*_1/2_ = 17.5 h; *F* = 80% for oral administration), particularly the considerably longer half-life and higher oral bioavailability in humans [24,25]. Further, tadalafil is mainly metabolized by CYP3A4 in humans, but dogs have different CYP isoforms and activities than humans [26]. In addition, it is well known that the metabolic rate of small animals is much faster than that of humans [27]. Therefore, this discrepancy may be attributable to the interspecies pharmacokinetic differences between humans and dogs.

The intranasal administration of PDE5 inhibitors for the treatment of ED has several potential advantages over the conventional oral routes. Among the intranasal administration methods, nasal sprays are better absorbed and have a higher bioavailability than nasal drops [28,29]. However, the disadvantages of intranasal administration must be considered, including nasal debris, inflammation, and nasal cavity anatomy alteration. In particular, nasal congestion is one of the mechanism-related adverse effects of PDE5 inhibitors [30], so a safety study will be required.

## 5. Conclusions

The pharmacokinetics of tadalafil were compared following the oral and intranasal administration of 5 mg tadalafil to dogs. The systemic exposures and absorption rates of tadalafil were significantly greater in the intranasal group, relative to those in the oral group. This study indicates the feasibility and benefits of developing a tadalafil nasal delivery system over the conventional oral route.

## Figures and Tables

**Table 1:** Noncompartmental pharmacokinetic parameters for the oral and intranasal administration of 5 mg tadalafil to dogs. Data are presented as the mean ± standard deviation (SD).

Parameter (Units)	Oral (*n* = 7)	Intranasal (*n* = 7)	*p* Value
*T_max_* (h)	1.71 ± 0.39	1.50 ± 0.41	0.337
*C_max_* (ng/mL)	59.49 ± 9.22	76.45 ± 12.07	*p* < 0.05
*AUC*_36*h*_ (ng·h/mL)	472.66 ± 102.70	771.10 ± 216.72	*p* < 0.01
*AUC_inf_* (ng·h/mL)	479.33 ± 102.88	790.23 ± 224.91	*p* < 0.01
*λ_z_* (h^−1^)	0.17 ± 0.04	0.13 ± 0.04	0.06
*t*_1/2_ (h)	4.17 ± 1.01	5.79 ± 1.47	*p* < 0.05
*Cl_t_/F* (L/h)	10.87 ± 2.43	6.98 ± 2.77	*p* < 0.05
*V_z_/F* (L)	64.13 ± 14.80	53.63 ± 5.07	0.101

*T_max_*—the time to reach *C_max_*; *C_max_*—peak plasma concentration; *AUC*_36*h*_—area under the plasma concentration-versus-time curve from time zero to 36 h; *AUC_inf_*—*AUC*_36*h*_ extrapolated to infinity; *λ_z_—* terminal elimination rate constant; *t*_1/2_—elimination half-life; *Cl_t_/F*—apparent total body clearance; *V_z_/F*—volume of distribution.

**Table 2:** Pharmacokinetic parameters for tadalafil estimated using a one-compartment model with first-order absorption and elimination constants for both the oral and intranasal administration of 5 mg tadalafil to dogs. Data are presented as mean ± standard deviation (SD).

Parameter (Units)	Oral (*n* = 7)	Intranasal (*n* = 7)	*p* Value
*K_a_* (h^−1^)	1.08 ± 0.11	1.53 ± 0.21	*p* < 0.001
*K_el_* (h^−1^)	0.18 ± 0.04	0.13 ± 0.04	*p* = 0.053
*V_c_/F* (L)	64.70 ± 8.41	54.83 ± 6.99	*p* < 0.05

*K_a_*—absorption rate constant; *K_el_*—elimination rate constant; *V_c_/F*—volume of distribution of tadalafil in the central compartment.
